# High-performance single-walled carbon nanotube transparent conducting film fabricated by using low feeding rate of ethanol solution

**DOI:** 10.1098/rsos.180392

**Published:** 2018-06-27

**Authors:** Er-Xiong Ding, Qiang Zhang, Nan Wei, Abu Taher Khan, Esko I. Kauppinen

**Affiliations:** Department of Applied Physics, Aalto University School of Science, Puumiehenkuja 2, 00076 Aalto, Espoo, Finland

**Keywords:** single-walled carbon nanotubes, transparent conducting films, ethanol, floating catalyst chemical vapour deposition

## Abstract

We report floating catalyst chemical vapour deposition synthesis of single-walled carbon nanotubes (SWCNTs) for high-performance transparent conducting films (TCFs) using low feeding rate of precursor solution. Herein, ethanol acts as carbon source, ferrocene and thiophene as catalyst precursor and growth promoter, respectively. By adopting a low feeding rate of 4 µl min^−1^, the fabricated TCFs present one of the lowest sheet resistances of *ca* 78 Ω sq.^−1^. at 90% transmittance. Optical characterizations demonstrate that the mean diameter of high-quality SWCNTs is up to 2 nm. Additionally, electron microcopy observations provide evidence that the mean length of SWCNT bundles is as long as 28.4 µm while the mean bundle diameter is only 5.3 nm. Moreover, very few CNT loops can be found in the film. Remarkably, the fraction of individual SWCNTs reaches 24.6%. All those morphology data account for the superior optoelectronic performance of our SWCNT TCFs.

## 1. Introduction

Single-walled carbon nanotubes (SWCNTs) have exceptional electrical and unique optical properties, rendering them being applied in various application fields, especially in transparent conducting films (TCFs) [1–4]. The fabrication methods of SWCNT TCFs are dominated by solution method [1,3,5] and dry-transfer method [2,6,7]. The dry-transfer method outperforms solution method in regard to retaining the primordial features of SWCNTs, because as-produced SWCNTs are collected at the outlet of a reactor using a membrane filter to form a thin film which can be press-transferred to a target substrate. Additionally, features like diameter [8], length [2] and bundle diameter [9] of SWCNTs can be controlled via adjusting growth parameters.

Specific applications of SWCNTs require particular diameters of SWCNTs. For instance, Asada *et al.* [10] have confirmed the superiority of small diameter SWCNTs for thin-film transistor applications in terms of both on/off ratio and carrier mobility. Theoretical and experimental studies indicate that the band gap of a semiconducting SWCNT (s-SWCNT) is inversely proportional to its diameter, following the relationship of *E*_g_ ∼ 0.6 eV/diameter (nm) [11,12]. Therefore, large diameter SWCNTs that feature narrow band gaps which induce excitation of a considerable number of carriers are preferable in the application of conductive films [13]. Besides, we have investigated the effect of mean bundle length of SWCNTs on the sheet resistance of TCFs, concluding that TCFs consisting of long nanotube bundles exhibit lower sheet resistances [2,9]. However, the mean bundle lengths of nanotubes in those works are shorter than 10 µm; longer nanotubes are highly needed to verify the finding. Another crucial factor affecting the conductivity of TCFs is the mean diameter of SWCNT bundle. Using conductance atomic force microscopy (AFM), Nirmalraj *et al.* [14] studied junction resistances in SWCNT networks and found that the junction between narrow nanotube bundles presents lower resistance. We also reported SWCNT TCFs with a low sheet resistance of 63 Ω sq.^−1^ at 90% transmittance via producing SWCNTs containing 60% individual ones [6]. In a floating catalyst chemical vapour deposition reactor, as-formed nanotubes in the gas phase undergo more or less mutual collisions due to Brownian diffusion, resulting in the formation of bundles. It is very likely that nanotube growth can be terminated by intertube collision, which restrains the growth of long nanotubes. Consequently, low feeding of the optimal combination of catalyst precursor and carbon source would produces narrow nanotube bundles with long length, due to low concentration of catalyst particles and carbon radicals. Our previous work [15] also demonstrated that lower feeding rate of precursors produces narrower SWCNT bundles, which needs to be verified by using further lower feeding rate. In short, narrow bundles consisting of large diameter and long SWCNTs are greatly needed for the direct fabrication of highly conductive SWCNT TCFs.

In this work, we adopted a very low feeding rate of precursors to produce long and narrow SWCNT bundles for TCF fabrication. The doped TCF presents a low sheet resistance of *ca* 78 Ω sq.^−1^ at 90% transmittance. Then, both macro properties and micro structure were explored to reveal the causes of the high conductivity. The excellent optoelectronic performance of SWCNT TCFs highlights great potential applications in flexible and transparent conductors, such as solar cells and organic light-emitting diodes (OLEDs).

## 2. Experimental procedure

### 2.1. Synthesis of single-walled carbon nanotubes

The experimental details and optimization process have been described elsewhere [15]. Briefly, 0.3 wt% ferrocene (98%, Sigma-Aldrich) and a small amount of thiophene (≥99%, Sigma-Aldrich, molar ratio of S/Fe = 0.2) were dissolved in ethanol (99.5%, Altia Oyj, Finland) followed by 2 min of ultrasonication to form a homogeneous solution which was injected with a syringe pump (New Era Pump Systems, NE-1000 series, USA) at a feeding rate of 4 µl min^−1^. The evaporated precursors in a heating line were carried by 600 standard cubic centimetre (sccm) gaseous mixture of H_2_ (99.999%, AGA, Finland) and N_2_ (vaporized from liquid nitrogen, and purified by an oxygen/moisture trap, OT3-4, Agilent). In order to visually illustrate the relevant causes when using the feeding rate of 4 µl min^−1^, a higher feeding rate of 8 µl min^−1^ was also employed with 800 sccm carrier gas, while all other parameters were kept constant. In both cases, the volume ratio of H_2_ to N_2_ was 1 : 1. SWCNTs were collected at the downstream of the reactor using a membrane filter (Merck Millipore, France, diameter of 25 mm, pore size of 0.45 µm) to form a thin film. The transparency of SWCNT film was controlled by collection time.

### 2.2. Doping of single-walled carbon nanotube films

SWCNT films on membrane filters were press-transferred to quartz slides (35 × 20 × 1 mm) followed by drop-cast wetting of 16 mM fresh AuCl_3_ (≥99.99% trace metals basis, Sigma-Aldrich) in acetonitrile (99.8%, Sigma-Aldrich) at room temperature in atmosphere. Five minutes later, the films were washed by pure acetonitrile for sheet resistance measurement after drying naturally. AuCl_3_ was selected as a dopant based on two reasons. The sheet resistances of AuCl_3_-doped SWCNT films are relatively stable at ambient conditions. Additionally, the intrinsic structure of SWCNTs could be preserved after AuCl_3_ treatment.

### 2.3. Measurement of sheet resistances

The sheet resistances of SWCNT films on quartz slides were measured with a Hewlett Packard 3485A multimeter four point probe system. The probe consists of four needles made of tungsten with spacing of 1 mm between each other. The needle has a radius of curvature of 100 µm and a loading force of 15 g each (60 g total). The reading number was multiplied by a factor of 4.532 to calculate the actual sheet resistance of SWCNT film. The presented sheet resistance value was averaged based on three measurements for each sample. The data points were nonlinearly fitted based on the method described in the literature [2].

### 2.4. Optical spectroscopy characterizations

A UV–visible–near-infrared spectrometer (Agilent Cary 5000) was employed to acquire optical absorption and transmittance spectra from SWCNT films on quartz slides in the sample beam path while a blank quartz slide was located in the reference beam path. The transmittance values were read from the spectra at 550 nm. Raman spectra were obtained using a Raman spectrometer (Horiba Jobin-Yvon Labram HR 800) equipped with excitation wavelengths of 488 nm, 514 nm and 633 nm. The displayed spectrum was averaged based on three spectra detected by each laser.

### 2.5. Microscopy characterizations

Sparsely distributed SWCNTs were collected onto SiO_2_/Si substrate by thermophoretic method [16] for measuring bundle length with scanning electron microscopy (SEM; Zeiss Sigma VP). In order to reduce charging effect, the microscope was operated at an acceleration voltage of 1 kV using an Inlens electron detector. Surface roughnesses of SWCNT films were acquired from AFM (Dimension 3100) scanning operated in tapping mode. SWCNT films on membrane filters were press-transferred to SiO_2_/Si substrates, followed by ethanol densification before AFM measurement. For transmission electron microscopy (TEM) observation, SWCNTs were directly deposited onto copper grid coated with holey carbon film. A JEOL 2200FS Double Cs-corrected TEM was employed for high-resolution imaging at 200 kV.

## 3. Results and discussion

**Figure 1.**
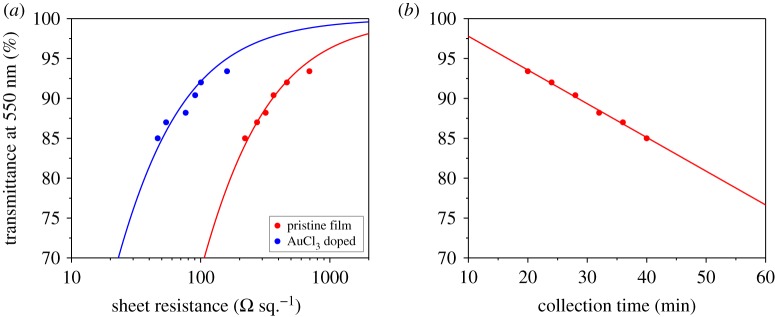
(*a*) Optoelectronic performance of SWCNT TCFs evaluated by the relationship of sheet resistance versus transmittance. (*b*) The relationship of collection time versus transmittance. Feeding rate is 4 µl min^−1^.

Compared with our previous report [15], only the feeding rate of precursor solution and the volume of carrier gas were adjusted in this work with other parameters kept constant. We found that feeding rate must be matched with appropriate volume of carrier gas in which both H_2_ and N_2_ play significant roles. Moreover, not only H_2_, but also N_2_ has a very narrow window for the fabrication of highly conductive SWCNT films. The optoelectronic results, at a feeding rate of 4 µl min^−1^, of the optimized combination of H_2_ and N_2_ are displayed in figure 1*a*. The pristine film (partially ambient-doped) exhibits a sheet resistance value of *ca* 360 Ω sq.^−1^ which drops to *ca* 78 Ω sq.^−1^ at 90% transmittance through AuCl_3_ treatment. Nevertheless, the collection time of 90 T% film with a diameter of 25 mm prolongs to 28 min (figure 1*b*). After optimizing growth parameters by the assessment of optoelectronic performance, samples were collected for optical spectroscopy and electron microscopy characterizations to figure out the reason behind the outstanding conductivity of SWCNT TCFs.

**Figure 2.**
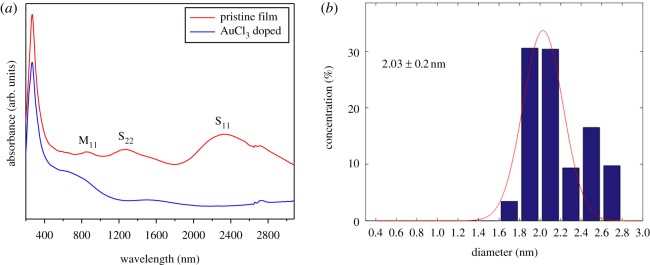
(*a*) Optical absorption spectra of SWCNT films. (*b*) Gaussian fitting result of diameter distribution (mean diameter ± standard deviation) of SWCNTs based on the absorption spectrum of pristine film in (*a*). Feeding rate is 4 µl min^−1^.

Optical absorption spectrum offers one route to check the macro properties of SWCNT films. Therefore, optical absorption spectra were firstly studied. The optical spectrum of SWCNTs is generally characterized by the van Hove singularity transitions $E_{11}^{S}$ and $E_{22}^{S}$ of s-SWCNTs and $E_{11}^{M}$ transition of metallic SWCNTs. These three characteristic peaks (S_11_ ∼ 2330 nm, S_22_ ∼ 1270 nm, M_11_ ∼ 850 nm) can be clearly seen in the spectrum of pristine film while S_11_ and S_22_ disappear through doping treatment of the pristine film by AuCl_3_ (figure 2*a*). AuCl_3_ doping initiates a charge transfer from SWCNTs to AuCl_3_, which gives rise to an increased work function of SWCNTs and a downshift of Fermi level towards valence band [17], modifying the van Hove singularity transitions of s-SWCNTs and vanishing S_11_ and S_22_ peaks correspondingly. Using the Matlab code developed by Tian *et al.* [18], the spectrum from pristine film displayed in figure 2*a* was fitted to acquire diameter distribution of SWCNTs. The fitting result concludes that the mean diameter of SWCNTs is around 2 nm, and more than 60% SWCNTs locate in the diameter range of 1.8 – 2.2 nm (figure 2*b*). The absorption spectrum of SWCNT film obtained at a higher feeding rate of 8 µl min^−1^, and corresponding fitting result are displayed in electronic supplementary material, figure S1. No apparent distinction of diameters can be found between two films with the variation of feeding rate. As mentioned above, large diameter s-SWCNTs possess narrow band gaps which facilitate electron hopping from valance band to conduction band, enhancing charge transport and improving conductivity of SWCNT films accordingly.

**Figure 3.**
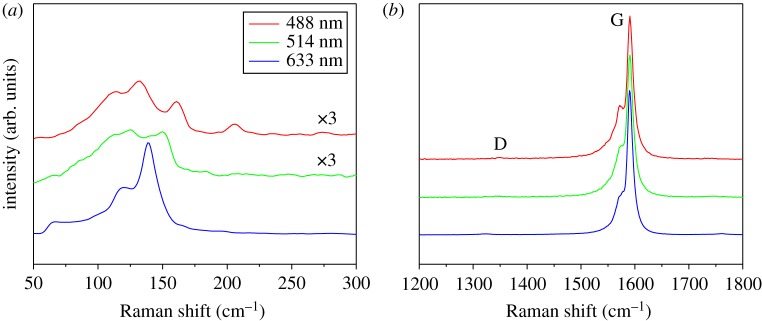
(*a*) RBM peaks, (*b*) G and D bands in Raman spectra of SWCNT films excited by 488 nm, 514 nm and 633 nm laser. Feeding rate is 4 µl min^−1^.

To estimate the quality of SWCNT films, Raman spectroscopy method was selected. The intensity ratio of graphitic (G) band (approx. 1590 cm^−1^) to disorder (D) band (approx. 1340 cm^−1^), *I*_G_/*I*_D_, in Raman spectrum is usually calculated to semi-qualitatively evaluate the quality of SWCNT films. Seen from figure 3*b*, the D bands are nearly invisible. Furthermore, *I*_G_/*I*_D_ values were calculated to be 45, 55 and 106 for 488 nm, 514 nm and 633 nm laser, respectively, indicating high-quality SWCNT films. The Raman spectra of SWCNT film obtained at feeding rate of 8 µl min^−1^ are shown in electronic supplementary material, figure S2. All the *I*_G_/*I*_D_ values are slightly lower than those from SWCNT film collected at lower feeding rate of 4 µl min^−1^. Likewise, the diameters of resonant SWCNTs can also be calculated from the frequencies of radial breathing mode (RBM) peaks (figure 3*a*) following the equation of $\omega_{\mathrm{RBM}}=A/d_{t}+B$, where A=217.8 and B=15.7 for film samples [18]. The majority of the diameters of resonant SWCNTs are in the range of 1.5 – 2.3 nm which is situated in the diameter range obtained from the fitting result of absorption spectrum.

**Figure 4.**
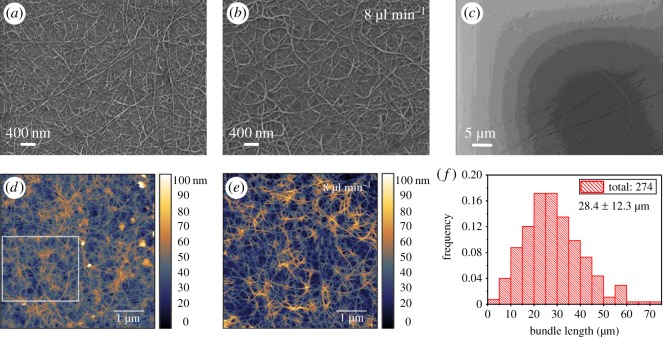
SEM micrographs of surface morphologies of ethanol-densified approximately 90 T% SWCNT films at feeding rates of (*a*) 4 µl min^−1^ and (*b*) 8 µl min^−1^. (*c*) SEM micrograph of SWCNT bundles on SiO_2_/Si substrate at feeding rate of 4 µl min^−1^. (*d*) AFM micrograph of SWCNT film (87 T%) at feeding rate of 4 µl min^−1^. Only the white square was considered for the analysis of RMS roughness, the white dots are Au particles due to the film used in this case being doped. (*e*) AFM micrograph of SWCNT film (88 T%) at feeding rate of 8 µl min^−1^. Whole area, i.e. 5 × 5 µm, was considered. (*f*) Length distribution of SWCNT bundles at feeding rate of 4 µl min^−1^. The numbers are mean length ± standard deviation.

In order to deeply explore the causes of excellent conductivity of SWCNT films, electron microscopy characterizations are needed to investigate the micro morphology and structure. Firstly, surface morphology of SWCNT film was checked using SEM. Figure 4*a* displays one SEM image of the surface morphology of an ethanol-densified SWCNT film. As a comparison, one SEM image of the morphology of the SWCNT film fabricated using a higher feeding rate of 8 µl min^−1^ is also shown in figure 4*b*. Compared with the morphology in figure 4*b*, far fewer CNT loops and narrower bundles are distinctly visible in figure 4*a*. Moreover, surface roughnesses of SWCNT films were also determined by AFM. The root-mean-square (RMS) roughness of SWCNT film obtained at feeding rate of 4 µl min^−1^ is only 9.2 ± 1.0 nm (figure 4*d*), while it increases to 15.1 ± 1.6 nm (figure 4*e*) for the film fabricated at 8 µl min^−1^, which is ascribed to the thicker CNT bundles. The AFM result is consistent with that from SEM observation. As only feeding rate and carrier gas were altered in this case, we consider that it is the decrease of volume of carrier gas that accounts for the reduction of number of CNT loops. Theoretical studies have proposed the existence of back flow at the outlet of a quartz tube inside a high-temperature furnace [19,20]. The back flow boosted by the phenomenon of thermophoresis can trigger local convection vortex which might be more obvious when using a high flow rate, bringing about many CNT loops. Additionally, nanotubes in a bundle can have different growth speeds, i.e. the inboard nanotube in a loop has lower growth speed while the outboard nanotube grows faster, which is similar to the formation mechanism of carbon nanocoils [21], promoting the formation of CNT loops as well. As was demonstrated by electrostatic force microscopy, CNT loops behave like confining barriers which can trap charge carriers inside, restraining the transportation of charge carriers among nanotubes [22]. Consequently, SWCNT film containing fewer CNT loops exhibits higher conductivity. In addition, the length of SWCNT bundle is another essential parameter that affects film conductivity. Sparsely distributed SWCNTs were directly collected on SiO_2_/Si substrate for SEM imaging (figure 4*c*) to statistically gather SWCNT bundles for measurement of bundle length. The histogram result shows a mean length of SWCNT bundles of 28.4 µm (figure 4*f*) which is significantly longer than those collected from carbon monoxide reactors [2,6,9], and slightly longer than that collected from ethanol reactor [15]. It was expected that the bundle length should have been even longer based on two phenomena. On the one hand, reduced volume of carrier gas increases the growth time of SWCNTs at elevated temperature in a quartz tube. On the other hand, lowered feeding rate decreases particle concentration in gas phase, which fates the mutual collision of as-formed tubes, extending the final length of nanotubes. Thus, it is supposed that insufficient counting of CNT bundles in our previous work [15] might somehow overestimate the actual length. SWCNTs produced using the higher feeding rate of 8 µl min^−1^ were also collected using the same method for length measurement, though the majority of CNT bundles are entangled even at a very low density (electronic supplementary material, figure S3). As a consequence, we failed to gather enough individual CNT bundles for a statistical study. It has been affirmed that the overall resistance of SWCNT networks is dominated by the resistances of contact junctions [14]. Especially, the resistance of the junction between semiconducting nanotubes and metallic counterparts contributes much more to the overall resistance in virtue of the existence of Schottky barrier. Therefore, SWCNT film comprised of long CNT bundles has fewer contact junctions, improving film conductivity as well.

**Figure 5.**
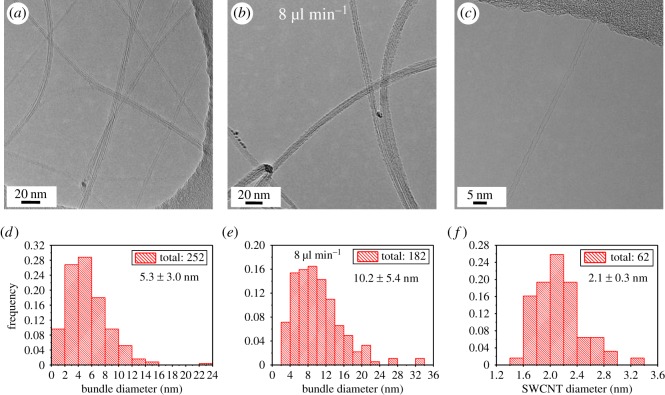
TEM micrographs of SWCNT bundles at feeding rates of (*a*) 4 µl min^−1^ and (*b*) 8 µl min^−1^. (*c*) High magnification TEM micrograph of an individual SWCNT at feeding rate of 4 µl min^−1^. Histogram distributions of bundle diameters at feeding rates of (*d*) 4 µl min^−1^ and (*e*) 8 µl min^−1^. (*f*) Diameter distribution of individual SWCNTs at feeding rate of 4 µl min^−1^. The numbers in (*d–f*) are mean diameter ± standard deviation.

TEM was used to reveal the structure of SWCNTs. SWCNTs are more or less bundled in an SWCNT network, and the diameters of bundles in a thin film are primarily determined by the bundles floating out from a reactor. Meanwhile, it should be pointed out that some bundles can re-bundle during film formation, owing to the attraction of van der Waals forces. Hence, a proper density of as-produced SWCNTs was deposited onto a TEM grid situated on a membrane filter to observe the diameters of SWCNT bundles. Similarly, SWCNTs produced with the higher feeding rate of 8 µl min^−1^ were collected as well for TEM imaging to better illustrate the origins of advanced conductivity. Several individual SWCNTs are visible in the TEM image in figure 5*a* which displays a few catalyst particles and small bundles. By statistically counting 252 bundles, we were able to calculate the mean diameter of SWCNT bundles to be 5.3 nm (figure 5*d*), while the mean bundle diameter at 8 µl min^−1^ is almost two times larger (figure 5*b*,*e*). Remarkably, it was found that individual SWCNTs account for 24.6% which is much higher than that from a higher feeding rate of precursors. Higher feeding rate leads to increased concentration of catalyst particles and carbon radicals which produce more nanotubes at a certain time, promoting mutual collisions to form bundles. The individual ones (figure 5*c*) were divided from the whole-counted counterparts and constitute a histogram shown in figure 5*f*. The diameter distribution of SWCNTs is nicely matched with that fitted from absorption spectrum. Further gathering of individual SWCNTs will minimize the discrepancy of mean diameters. The individual SWCNTs together with those narrow bundles greatly enhance conductivity of film, which has been experimentally and theoretically verified by our previous works using highly individual SWCNTs for thin-film studies [6,9,23]. They proposed that conduction paths would increase at a given optical transparency in a film containing more individual tubes, because current transport mainly occurs on the surface of bundles [23].

**Figure 6.**
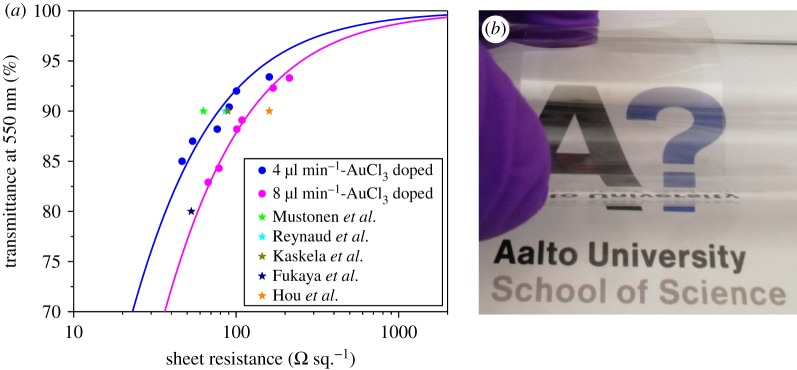
(*a*) Comparison of optoelectronic performance of SWCNT TCFs with some recently reported ones. (*b*) Photograph of a 25 mm SWCNT film on PET substrate.

Finally, the optoelectronic performance of the doped SWCNT TCFs in this work was compared with some recently reported values [6,9,24–26] for those fabricated by the same dry-transfer method. We found that the performance of our SWCNT TCFs fabricated at low feeding rate of 4 µl min^−1^ is superior to that of most of the reported films (figure 6*a*). The SWCNT TCFs fabricated at higher feeding rate of 8 µl min^−1^ show a sheet resistance value of *ca* 123 Ω sq.^−1^ at 90% transmittance, meaning a 36.6% improvement of film conductivity caused by lowering the feeding rate. Mustonen *et al.* [6] reported a low sheet resistance of 63 Ω sq.^−1^ at 90% transmittance, though the collection time, even for a 13 mm film, is rather long, which is not applicable for large-scale fabrication. A 25 mm film near 90 T% was transferred to polyethylene terephthalate (PET) substrate to demonstrate the transparency and flexibility of SWCNT TCF (figure 6*b*).

## 4. Conclusion

To conclude, we have demonstrated that using a low feeding rate of precursors can produce long and narrow bundled SWCNTs for fabrication of highly conductive TCFs from a floating catalyst chemical vapour deposition reactor. After AuCl_3_ doping, the TCFs exhibit a low sheet resistance of *ca* 78 Ω sq.^−1^ at 90% transmittance. The enhanced conductivity was elucidated in terms of SWCNT diameter and micro morphology. The large diameter SWCNTs possessing narrow band gaps intrinsically contribute a high concentration of charge carriers. Besides, the conductivity is strongly related to CNT loops and bundle length as well as bundle diameter. More straight and long CNT bundles will decrease the transportation barriers of charge carriers, improving conductivity of SWCNT film. Small CNT bundles and a high fraction of individual tubes will provide increased number of conduction pathways. In summary, the micro morphology of CNT bundles can be controlled by tuning the feeding rate of precursor solution. Fewer CNT loops and higher fraction of individual SWCNTs are mainly responsible for the improved conductivity using a low feeding rate in this work. The excellent optoelectronic performance of demonstrated SWCNT TCFs indicates their potential applications in solar cells, OLEDs, and further increases the likelihood to replace indium tin oxide film.

## Supplementary Material

Figures S1 - S3

## Data accessibility

All the data are available from the Dryad Digital Repository: http://dx.doi.org/10.5061/dryad.kp1hk40 [27].
