# Supplementary material for: High-performance single-walled carbon nanotube transparent conducting film fabricated by using low feeding rate of ethanol solution
Source: R Soc Open Sci. 2018 Jun 27;5(6):180392. doi: 10.1098/rsos.180392 (PMC6030293; doi:10.1098/rsos.180392)
Supplement: Figures S1 - S3 [file rsos180392supp1.docx]

**Royal Society Open Science**

**Supporting information for:**

**High performance single-walled carbon nanotube transparent conducting film fabricated by using low feeding rate of ethanol solution**

Er-Xiong Ding, Qiang Zhang, Nan Wei, Abu Taher Khan and Esko I. Kauppinen^[[1]](#footnote-2)^*

Department of Applied Physics, Aalto University School of Science, Puumiehenkuja 2, 00076 Aalto, Espoo, Finland.

**Figure S1.** (a) Optical absorption spectrum of pristine SWCNT film. (b) Gaussian fitting result of diameter distribution (mean diameter ± standard deviation) of SWCNTs based on the absorption spectrum in (a). Feeding rate is 8 μl/min.

**Figure S2.** (a) RBM peaks, (b) G and D bands in Raman spectra of SWCNT film excited by 488 nm, 514 nm and 633 nm laser. I_G_/I_D_ values were calculated to be 42, 52 and 95 for 488 nm, 514 nm and 633 nm laser, respectively. Feeding rate is 8 μl/min.

**Figure S3.** SEM micrograph of SWCNT bundles on SiO_2_/Si substrate at a feeding rate of 8 μl/min. The nanotube bundles are entangled.

1. E-mail address: [esko.kauppinen@aalto.fi](mailto:esko.kauppinen@aalto.fi) [↑](#footnote-ref-2)
